# Absorption, tissue distribution, and excretion of glycycoumarin, a major bioactive coumarin from Chinese licorice (*Glycyrrhiza uralensis* Fisch)

**DOI:** 10.3389/fphar.2023.1216985

**Published:** 2023-07-07

**Authors:** Linhu Ye, Lei Cheng, Yan Deng, Sen Wang, Xinyu Wu, Shuiping Ou, Qi Chang, Xinqian Zhao, Wen Zhou, Jinghua Yu, Zuqiang Wu

**Affiliations:** ^1^ The Sixth Affiliated Hospital, School of Medicine, South China University of Technology, Nanhai District People’s Hospital of Foshan, Foshan, China; ^2^ Bijie City First People’s Hospital, Bijie, China; ^3^ School of Pharmacy, Zunyi Medicinal University, Zunyi, China; ^4^ Institute of Medicinal Plant Development, Chinese Academy of Medical Sciences and Peking Union Medical College, Beijing, China

**Keywords:** glycycoumarin, pharmacokinetics, bioavailability, tissue distribution, LC-MS/MS

## Abstract

Licorice (*Glycyrrhiza uralensis* Fisch) is a natural plant resource widely used as a food and herbal medication in China. Glycycoumarin (GCM) is a major coumarin in licorice that possesses several biological activities. However, little is known about its pharmacokinetic profile. The present study aimed to describe the oral absorption, tissue distribution, and excretion of GCM in rats. Free (parent drug) and/or total (parent drug plus the glucuronidated metabolite) GCM in biological samples was quantified before and after the hydrolysis reaction with *β*-glucuronidase using a reliable LC-MS/MS method. The results indicated that GCM was rapidly absorbed and transformed into its conjugated metabolites after administration. Free GCM plasma concentrations after *i. v.* (10 mg/kg) administration quickly decreased with an average t_1/2,λz_ of 0.71 h, whereas the total GCM concentration reduced slowly with a t_1/2, λz_ of 2.46 h. The area under the curve of glucuronidated metabolites was approximately four-times higher than that of free GCM. Presumably, because of hepatic and/or intestinal tract first-pass metabolism, GCM exhibited a poor bioavailability of 9.22%, as estimated from its total plasma concentration. Additionally, GCM was distributed rapidly and widely in various tissues except the brain. The liver had the highest concentration; further, GCM was promptly eliminated from test tissues after intraperitoneal (20 mg/kg) administration, but only a small amount of GCM was excreted via bile and urine. Overall, GCM is absorbed and rapidly transformed into its conjugated metabolites with low bioavailability; further, it is distributed in various tissues, except the brain. These pharmacokinetic results are helpful for better understanding the characteristics and pharmacological effects of GCM.

## 1 Introduction

Licorice (*Glycyrrhiza uralensis* Fisch), known as Gan Cao in traditional Chinese medicine, is one of the best-known herbal medicines throughout Asia and is widely used as a medicine and food in China. Licorice appears in the pharmacopoeia of the People’s Republic of China, where its applications include detoxification and treatment of spleen and stomach disorders, palpitations and shortness of breath, coughs, influenza infection, and liver disease. Pharmacological studies have demonstrated that licorice exhibits various biological activities, including antioxidant, anti-inflammatory, antidiabetic, anticancer, and skin-whitening effects (Asl and Hosseinzadeh, 2008; Fu et al., 2013; Fukuchi et al., 2016). Phytochemical studies have shown that licorice is rich in triterpenes and their glycosides, flavonoids, and coumarins (Ji et al., 2016).

**FIGURE 1:**
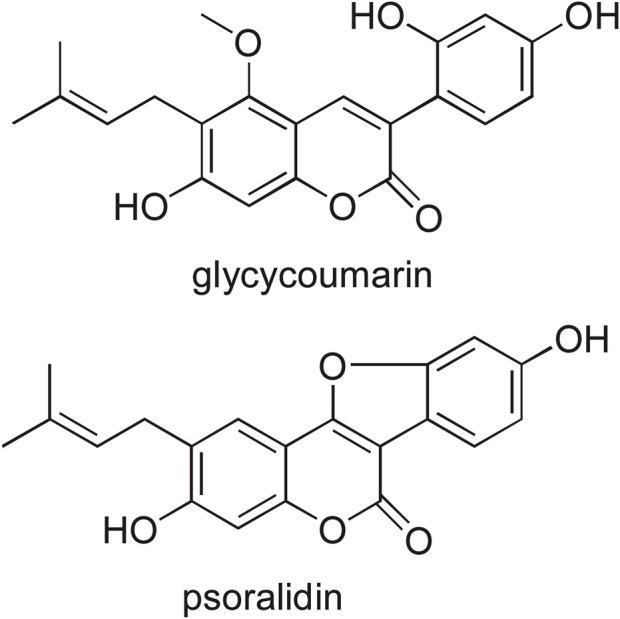
Chemical structure of glycycoumarin and psoralidin (internal standard).

Glycycoumarin (GCM, Figure 1) is a major coumarin in licorice, accounting for 0.81 mg/g in the crude roots and rhizomes of licorice (Qiao et al., 2014b). The reported biological activities of GCM include antioxidant, anti-inflammatory and anti-hepatitis C virus activities (Adianti et al., 2014). More recently, additional beneficial effects of GCM have been described. For example, GCM exerts anti-hepatoma effects by binding to and inactivating the oncogenic kinase TOPK and activating the p53 pathway (Song et al., 2016). It also improves the efficacy of BH3 mimetic ABT-737 against liver cancer and attenuates the platelet toxicity of ABT-737 (Zhang et al., 2018). Moreover, GCM can ameliorate alcohol-induced hepatic injury by activating Nrf2 and autophagy (Song et al., 2015) and protect against acetaminophen-induced liver injury by activating sustained autophagy (Yan et al., 2018). The major metabolic pathways of GCM *in vivo* are glucuronidation and hydroxylation; three metabolites are formed in the glucuronidation reaction, and the binding sites may include its three hydroxyl groups (Wang et al., 2014).

Despite increasing studies on the biological activities of GCM and its underlying metabolic pathways, little is known about its pharmacokinetic profile. In this study, we performed a rapid and reliable liquid chromatography coupled with mass spectrometry (LC-MS/MS) method for determining GCM abundance. This method was successfully used to assess the pharmacokinetic properties of GCM and its tissue distribution in rats. To our knowledge, this study is the first to report the pharmacokinetics of purified GCM, including its oral absorption, bioavailability, tissue distribution, and excretion, after oral, intravenous (*i.v*.), and intraperitoneal administration in rats for exploring its disposal in the body and further understand its *in vivo* pharmacological activities.

## 2 Materials and methods

### 2.1 Chemicals and reagents

Glycycoumarin (purity>98%, Cat# DSTDG010801) was used as a reference compound and was purchased from Lemeitian Medical Technology Co., Ltd. (Chengdu, China). Psoralidin (Cat# 180526), used as an internal standard (IS), was obtained from Ronghe Pharmaceutical Sciences (Shanghai, China). *β*-Glucuronidase (Type H-1, Cat# SLCH4420) was obtained from Sigma (St. Louis, MO, United States). Solutol HS-15 (Cat# 159186) was obtained from MedChemExpress LLC (Shanghai, China). Anhydrous sodium acetate (Cat# C12095781) was purchased from Macklin Biochemical Technology Co., Ltd. (Shanghai, China). HPLC-grade acetonitrile and methanol were purchased from Fisher Scientific (Emerson, IA, United States). MCI GEL was purchased from Mitsubishi Chemical (Tokyo, Japan). Silica gel was obtained from Qingdao Haiyang Chemical Co., Ltd. (Qingdao, China). Analytical-grade ethyl acetate, methanol, and dichloromethane were obtained from Fuyu Fine Chemical Co., Ltd. (Tianjin, China).

### 2.2 Plant material

The roots and rhizomes of licorice (*G. uralensis* Fisch.) were obtained from the Institute of Medicinal Plant Development, Chinese Academy of Medical Sciences, and Peking Union Medical College in November 2018 in Beijing. It was identified by Associate Professor Cha Qin from the Institute of Chinese Materia Medica of Bijie City. A voucher specimen (No. 2018-1101) was deposited at the Key Laboratory of Pharmacy of Bijie City First People’s Hospital, Guizhou, China.

### 2.3 Extraction and isolation of glycycoumarin

Licorice (21 kg) was powdered and extracted thrice with 90% (v/v) ethanol for 2 h under reflux. The extracts were pooled and filtered, concentrated *in vacuo* to remove ethanol, and free-dried to obtain a dry licorice alcohol extract (3.6 kg). The licorice extract was dissolved in distilled water and extracted using ethyl acetate. The resulting ethyl acetate extract (860 g) was applied to a silica gel column and successively eluted with 0, 5, 10, 30, 50, and 100% (v/v) methanol in dichloromethane. The eluents from the 5% methanol application were collected and subjected to MCI GEL column chromatography for decolorization. The extracted compounds were then separated by repeated column chromatography and preparative high-performance liquid chromatography (HPLC) to isolate GCM. The chemical structure of the isolated GCM was identified by comparing the mass and fragmentation profiles obtained by electrospray ionization mass spectrometry (ESI-MS), as well as nuclear magnetic resonance (^1^H- and ^13^C-NMR) spectra (Bruker AV-600, Fällanden, Switzerland) with those available in the literature. We also compared the GCM HPLC retention times and online UV spectra with those of the reference compound. The purity of GCM was assessed using HPLC-UV analysis.

### 2.4 Animals

Male Sprague-Dawley rats (200 ± 20 g) were obtained from SJA Laboratory Animal Co. Ltd. (Hunan, China). All animals were kept under standard conditions of light, humidity, and temperature, and were allowed free access to a standard diet and water. All animal experiments were approved by the Animal Ethics Committee of Bijie City First People’s Hospital (BY[2019]-02-198). On the day before GCM administration, rats were subjected to a minor surgical procedure. Briefly, a polyurethane catheter (0.59 mm ID, 0.94 mm OD; Skillsmodel Limited, Beijing, China) was cannulated into the right jugular vein while under anesthesia induced using an intraperitoneal dose of chloral hydrate at 350 mg/kg. After surgery, the rats were individually placed in metabolism cages to allow recovery for at least 24 h. The rats were fasted overnight with free access to water before GCM administration.

### 2.5 Preparation of standards and quality control samples

GCM (2.0 mg) and IS (2.0 mg) were accurately weighed and then dissolved in methanol to prepare respective stock solutions with a concentration of 2 mg/mL each. After dilution with methanol, the stock solution was prepared as a series of standard working solutions. The working solutions were used to spike the plasma, bile, urine, or tissue homogenates to obtain final GCM concentrations of 1, 2, 5, 10, 20, 50, 100, 200, 500, 1000, and 2000 ng/mL for the standard curve of all samples. The final concentration of IS used in the plasma, bile, urine, and tissue homogenates was 100 ng/mL. Four concentrations (5, 15, 150, and 1500 ng/mL) of quality control (QC) samples were freshly prepared for method validation.

### 2.6 Drug administration and sample collection

#### 2.6.1 Drug administration

All GCM dosing drugs were freshly prepared at a concentration of 10 mg/mL prior to the experiments. Briefly, GCM was dissolved in normal saline containing 5% (v/v) Solutol HS 15. The animals were randomly divided into three groups. The *i. v.* administration group was treated intravenously with 10 mg/mL GCM by quick injection through the cannulated catheter, and approximately 0.2 mL saline with 20 units of heparin was injected to flush the catheter and prevent blood coagulation. The oral administration group was treated with 20 mg/mL GCM in a single gastric gavage dose, and the intraperitoneal injection group received 20 mg/kg GCM through intraperitoneal injection.

#### 2.6.2 Plasma sample collection

Approximately 0.3 mL blood samples were collected via the cannula and placed into heparinized micro centrifuge (Eppendorf) tubes at multiple time-points (0.08, 0.25, 0.50, 1, 2, 3, 4, 5, 6, 7, 8, 10, 12, and 24 h) after GCM administration. The samples were centrifuged at 3000 × *g* for 5 min at 4°C for plasma separation; a 0.1 mL aliquot of each separated plasma sample was harvested and stored at −40°C until assessed.

#### 2.6.3 Tissue sample collection

Twenty-five rats were randomly divided into five groups and administered 20 mg/kg GCM intraperitoneally. Samples of various tissues (heart, liver, spleen, lung, kidney, and brain) of a certain weight were collected from the rats at 0.5, 1, 2, 4, and 6 h following GCM administration. All tissues were then rinsed with normal saline to clean the blood, blotted with filter paper, and stored at −40°C until use. Blood samples were collected *via* the carotid artery at the specified time points, and plasma samples were harvested as described above.

#### 2.6.4 Bile sample collection

Rats (n = 6) underwent bile fistula cannulation under anesthesia prior to bile sample collection. Bile samples from rats following *i. v.* GCM administration (10 mg/kg) were collected *via* cannula every 2 h over an 8 h collection period. Samples were collected from the catheter and delivered into acidified (0.1% HCl) centrifuge tubes. The rats were placed on thermostatic plates and kept under mild anesthesia throughout the experiment. The bile samples were diluted with saline and stored at −40°C until assayed.

#### 2.6.5 Urine sample collection

Six rats were individually placed in metabolic cages and administered 10 mg/kg GCM by *i. v.* injection. Urine samples were collected over 24 h after administration in a urine reservoir containing 2 mL 0.1% HCl to avoid GCM degradation. Each urine reservoir was rinsed with 5 mL normal saline after collection. Every urine sample was diluted to 20 mL with normal saline and then stored at −40°C until assayed.

### 2.7 Determination of GCM levels in biological samples

Free (parent drug) and/or total (including parent and glucuronidated metabolites) GCM concentrations in rat plasma, tissues, urine, and bile were quantitatively assayed using LC-MS/MS before and after hydrolytic treatment with *β*-glucuronidase.

#### 2.7.1 Preparation of plasma samples

Aliquots (100 μL) of plasma samples were divided into two equal fractions for determining the free and total GCM concentrations, as previously described (Chang et al., 2012). Briefly, two aliquots of 50 μL plasma were incubated at 37°C with or without *β*-glucuronidase at a concentration of 1,200 units for total and free GCM, respectively. After 40 min of incubation, 10 μL of IS (1,000 ng/mL) was added to the mixtures followed by extraction with ethyl acetate. After centrifugation, the clear ethyl acetate layer was transferred and concentrated using a freeze-dry concentration method at 4°C. Subsequently, the remaining material was reconstituted in 200 μL of 90% (v/v) methanol. A 10 μL aliquot of the supernatant was injected into the LC-MS/MS system for analysis.

#### 2.7.2 Preparation of tissue samples

Tissue samples were accurately weighed and homogenized in ice-cold saline (1:4, w/v) using a glass tissue homogenizer. Homogenized organs were centrifuged at 12,000 × *g* for 10 min to obtain clear supernatants; the supernatant was then transferred to a clean test tube kept at −40°C until assayed.

#### 2.7.3 Preparation of gastrointestinal tract solutions

The gastrointestinal tract solutions were collected as described previously (Kong et al., 2013). Briefly, rats were fasted overnight and sacrificed; the entire stomach, small intestine, and colon of each rat were removed, and their contents were rapidly collected on ice. Each of the contents was suspended in buffer solutions at pH 1.2, 6.8, and 7.4. Following mixing and centrifugation, the protein content of the gastrointestinal tract solutions was assayed using the BCA protein assay kit from Solarbio (Beijing, China) and then adjusted to 12 mg/mL for further study.

#### 2.7.4 Preparation of bile and urine samples

Two aliquots of 50 μL prepared bile or urine samples were spiked with or without *β*-glucuronidase to determine the total or free GCM concentrations, as described above. The mixtures were incubated at 37°C for 40 min, and the mixtures were then spiked with 120 μL of ice-cold methanol containing IS to stop incubation. A 10 μL supernatant of each sample was used for the analysis.

### 2.8 Analysis of GCM stability in the gastrointestinal tract

The stability of GCM in the gastrointestinal tract, including chemical stability and enzymatic stability, was assessed using an *in vitro* method as described previously (Kong et al., 2013). Briefly, GCM was spiked into different pH (1.2, 6.8, and 7.4) buffers and gastrointestinal content solutions containing the GCM at a final concentration of 0.8 μg/mL. The solutions were then mixed and incubated at 37°C. Aliquots of 200 μL samples were collected at 0.5, 1, 2, and 4 h, and 200 μL ice-cold methanol containing 200 ng/mL IS was added to stop the incubation. After centrifugation, 10 μL of the supernatant was used for analysis. Each experiment was performed in triplicate.

### 2.9 LC-MS/MS conditions

An LC-MS/MS system comprising an Agilent 1260 HPLC system (Palo Alto, CA, United States) and an Applied Biosystems 4500 Q-Trap mass spectrometer equipped with an electrospray ionization source (Foster City, CA, United States) was used. Chromatographic separation was performed on a C18 column (100 × 4.6 mm, 3 μm, Thermo Fisher Scientific, Waltham, MA, United States) maintained at 30°C. The mobile phases comprised 0.1% (v/v) formic acid in water (A) and acetonitrile (B) at a flow rate of 0.4 mL/min. Phase B was linearly increased from 40% to 90% over a period of 0.1 min, maintained at 90% for 2 min, and then decreased to 40% for re-equilibration. The total run time was 4 min. For mass spectrometry detection, the positive ion mode was used and the ion spray voltage was set at 5,500 V. The operating conditions were as follows: ion source temperature, 450°C; curtain gas, 20 psi; collision gas, medium. Ion source gases 1 and 2 were both at 60 psi. The collision energies were 37 and 42 V, and the declustering potentials for GCM and IS were 110 and 136 V, respectively. Quantification was performed by multiple reaction monitoring (MRM) of molecular ions and product ions at *m/z* 369.3 and 284.7 for GCM and 337.0 and 280.9 for IS (Supplementary Figure S1). Analyst^®^ software (Version 1.6.3) was used for data acquisition and peak integration.

### 2.10 Pharmacokinetic data analysis

Pharmacokinetic parameters were calculated based on changes in the plasma drug concentration *versus* the time profile of each rat using a non-compartmental model using WinNonlin software (Pharsight Corporation, Mountain View, CA, United States, Version 6.1). Plasma maximum concentration (Cmax) and the time of Cmax (Tmax); initial plasma concentration (C_0_) for *i. v.* administration; terminal elimination half-life (t_1/2, λz_); area under the plasma concentration *versus* time curve from time zero to the last sample collection time point (AUC_0–t_) and to infinity (AUC_0–∞_); total body clearance (CL); and volume of distribution (V_d, λz_). Absolute bioavailability (F) was calculated based on the AUC_0–∞, oral_/AUC_0–∞,__*i. v.*_at equivalent doses.

## 3 Results and discussion

### 3.1 Isolation and identification of glycycoumarin

After several purification steps (silica gel column, MCI GEL™ column, and reverse-phase HPLC), pure GCM was isolated. Its chemical structure was characterized based on the high-resolution ESI-MS (HRESI-MS), ^1^H-NMR, and ^13^C-NMR spectra (Supplementary Figures S2–S5, Supplementary Material) in accordance with the literature (Ji et al., 2016). Furthermore, the HPLC retention time and online UV spectrum of the isolated GCM were the same as those of the reference standard. The purity of the isolated GCM was determined to be 97.4% using HPLC-UV analysis.

### 3.2 Method validation

Before the pharmacokinetic studies, a LC-MS/MS method was established and validated for the quantitative analysis of free and total GCM concentrations in rat plasma, urine, bile, and tissues.

#### 3.2.1 Specificity

**FIGURE 2:**
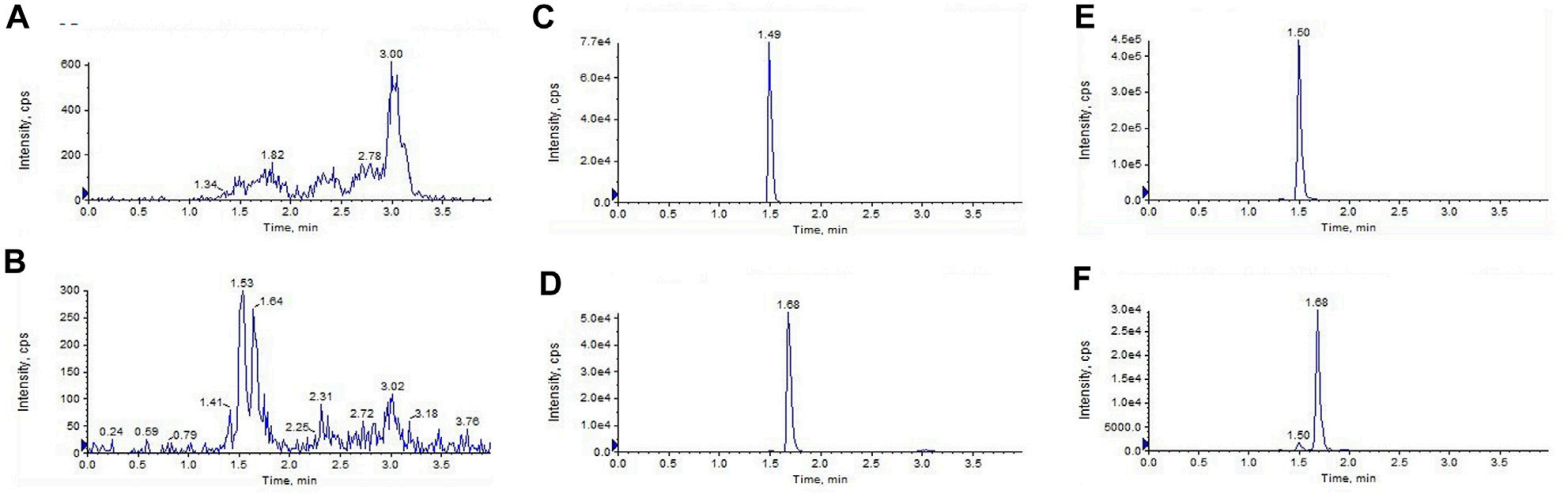
Liquid chromatography tandem-mass spectrometry chromatograms of **(A,B)**blank plasma **(C,D)** blank plasma spiked with glycycoumarin and the internal standard(IS) both at 100 ng/mL, and **(E,F)** the plasma sample 1 h after *i. v.* administration of glycycoumarin at a single dose of 10 mg/kg and spiked with IS.

Figure 2 shows the representative LC-MS/MS chromatograms of the blank plasma (Figures 2A, B), quality control plasma samples (blank plasma supplemented with GCM and IS at 100 ng/mL, respectively. Figures 2C, D), and plasma samples collected 1 h after *i. v.* dosing (10 mg/kg) of GCM (Figures 2E, F). The retention times of GCM and IS were 1.49 and 1.68 min, respectively. Supplementary Figure S6 shows the typical MRM chromatograms of pooled blank urine and bile samples spiked with GCM and IS; these were eluted at 1.49 and 1.68 min, respectively. The typical chromatograms of blank tissue homogenates spiked with GCM and IS were obtained under the same conditions (Supplementary Figure S7). This revealed that GCM and IS were completely separated, and that there was no interference to influence GCM quantification in the biological sample.

#### 3.2.2 Linearity, LLOD, and LLOQ

The standard calibration curves for rat plasma, urine, and bile containing GCM exhibited good linearity (*R* ^2^ > 0.99) over a concentration range of 2–2,000 ng/mL. The LLOD was 1 ng/mL, and the LLOQ was 2 ng/mL. Linear responses of GCM were observed at 5–2,000 ng/mL in the tissue samples. The LLOD was 1 ng/mL, and the LLOQ was 5 ng/mL. The results of the linear regression analyses are listed in Supplementary Table S1.

#### 3.2.3 Extraction recovery and matrix effect

The average extraction recovery of the QC samples ranged from 85.47% to 111.54% for GCM in all biological samples. The internal standard normalized matrix factor (ISNMF), which was evaluated by the matrix factor of the analyte *versus* the internal standard matrix factor, was recommended for evaluating the matrix effects (De Nicolò et al., 2017). The RSD of ISNMFs ranged from 1.75% to 14.14%, indicating that the influence of co-eluted substances could be corrected by using the IS.

#### 3.2.4 Stability, precision, and accuracy

Stability studies were carried out by analyzing different concentrations for QC after biological sample preparation at 4°C for 24 h and storage at −40°C for 7 days, respectively. As shown in Supplementary Table S2, their accuracies ranged from 85.53% to 114.13%, and all RSDs were within 14%.The intra-day and inter-day precision and accuracy for GCM are shown in Supplementary Table S3. The intra-day and inter-day precision of GCM determinations were <15%, and their accuracy ranged from 85.69% to 118.42% at 5, 15, 150, and 1,500 ng/mL.

#### 3.2.5 Dilution

The accuracy of dilution for 10,000 ng/mL GCM (n = 5) ranged from 86.24% to 112.28% in the plasma, heart, liver, and spleen. These values correspond to the Food and Drug Administration (FDA) criteria for bioanalytical validation methods.

### 3.3 Pharmacokinetics

**FIGURE 3:**
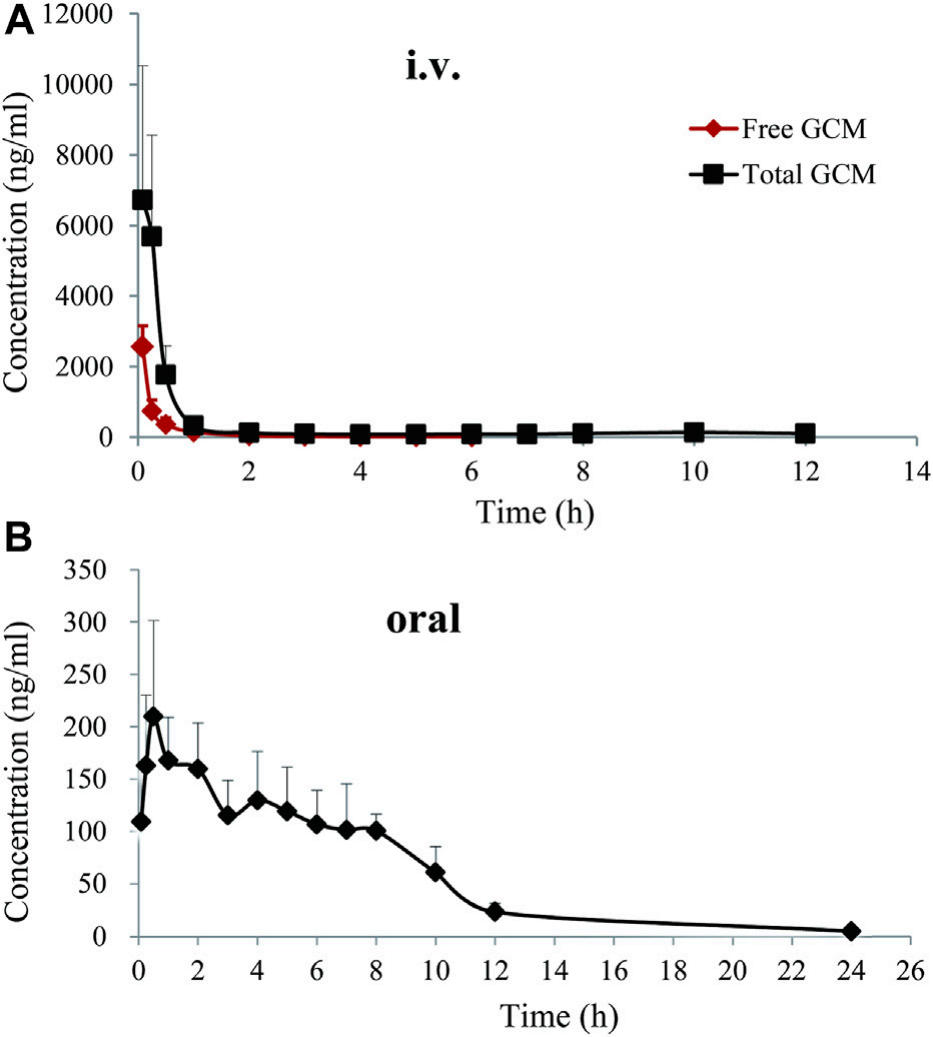
Free and total plasma concentration-time profiles of glycycoumarin in rats after single intravenous (*i.v*., 10 mg/kg)or oral administration (20 mg/kg). Each pointrepresentsthe mean ± standard deviation (n = 6).

To better understand the oral absorption and disposal process of GCM in the body, free and total plasma GCM concentrations (the parent form and glucuronidated metabolites) were determined. The plasma concentration *versus* time profiles of free and/or total GCM in rats after receiving an *i. v.* dose (10 mg/kg) or oral administration (20 mg/kg) are shown in Figure 3. The pharmacokinetic parameters of the free and/or total GCM are presented in Table 1. After *i. v.* dosing, only a minor quantity of parent GCM was detected in plasma samples after the first 6 h post-administration; it was almost undetectable in plasma collected after 6 h. It might be transformed into its glucuronidated metabolites because relatively high GCM concentrations were detected in the plasma after enzymatic treatment with *β*-glucuronidase. The results showed that the AUC_0-∞_ of conjugated GCM was approximately four times higher than that of free GCM. This implies that glucuronidated metabolites are the major form of GCM in rats. The glucuronidated metabolites were eliminated slowly from the body, with average t_1/2, λz_ values of 2.46 h after *i. v.* administration, more than 3 folds those of free GCM.

**TABLE 1:** Pharmacokinetic parameters of glycycoumarin (GCM) after a single intravenous (i.v., 10 mg/kg) or oral (20 mg/kg) administration to rats. All data are expressed as the mean ± standard deviation (n = 6).

Parameters	i.v. (10 mg/kg)		Oral (20 mg/kg)
	Free GCM	Total GCM	Total GCM
C_max_ (ng/mL)	-	-	232.18 ± 78.30
T_max_ (h)	0	0	0.79 ± 0.64
C_0_ (ng/mL)	5686.95 ± 1362.90	9727.38 ± 5068.72	-
t_1/2_, _λz_ (h)	0.71 ± 0.11	2.46 ± 0.61	4.75 ± 0.46
AUC_0–t_ (ng h/mL)	1163.66 ± 283.73	5033.80 ± 1840.97	983.23 ± 217.32
AUC_0–∞_(ng h/mL)	1169.64 ± 283.36	5521.77 ± 2112.90	1017.85 ± 238.94
CL/F (L/h/kg)	8.95 ± 2.0	-	-
V_d, λz_/F	9.25 ± 3.13	-	-
F (%)	-	-	9.22
Urine recovery (% of dose)			
0–12 h	0.28 ± 0.25	0.74 ± 0.46	
12–24 h	0.11 ± 0.10	0.23 ± 0.22	
Bile recovery (% of dose)			
0–2 h	0.20 ± 0.12	1.97 ± 0.59	
2–4 h	0.08 ± 0.06	1.71 ± 0.55	
4–6 h	0.05 ± 0.04	1.51 ± 0.38	
6–8 h	nd	1.33 ± 0.96	

“–” not applicable; “nd” not detectable.

After oral administration of 20 mg/kg, free GCM was almost undetectable in the plasma after 2 h. However, after treatment with *β*-glucuronidase, GCM was detected in the plasma over 24 h after dosing. Total GCM reached a C_max_ of 232.18 ng/mL at a T_max_ of 0.79 h, and was eliminated from the body with a t_1/2, λz_ of 4.75 h; the AUC_0-∞_ was 1,017.85 ng h/mL.The absolute bioavailability of GCM was not determined because the concentration of free GCM after oral administration was too low to be detected at most time points. The bioavailability of total GCM was estimated to be 9.22%, which was calculated by an AUC_0-∞_ of total GCM. This result suggested that a certain amount of GCM could be absorbed into the body and then quickly metabolized into glucuronidated metabolites *via* intestinal and/or hepatic first-pass effects.

GCM is reported to be rapidly absorbed into circulation and is involved in Phase I and Phase II biotransformation; further, GCM glucuronidation and hydroxylation are the main metabolic pathways involved (Wang et al., 2014). The isoprenyl methyl group is the major metabolic participant in hydroxylation reactions. The glucuronidation products include the parent GCM and its hydroxyl metabolites conjugated to glucuronic acid. However, in this study, our developed method could only monitor GCM but not detect other metabolites. Cytochrome P450 enzymes are also reported to be involved in GCM metabolism because many GCM metabolites have been demonstrated to be catalyzed in rat liver microsomes (Wang et al., 2014). Thus, the most likely reason for the low oral bioavailability of GCM might be related to its extensive first-pass effect when transiting the liver and/or intestine, which deserves further study.

### 3.4 *In vitro* stability of GCM

**FIGURE 4:**
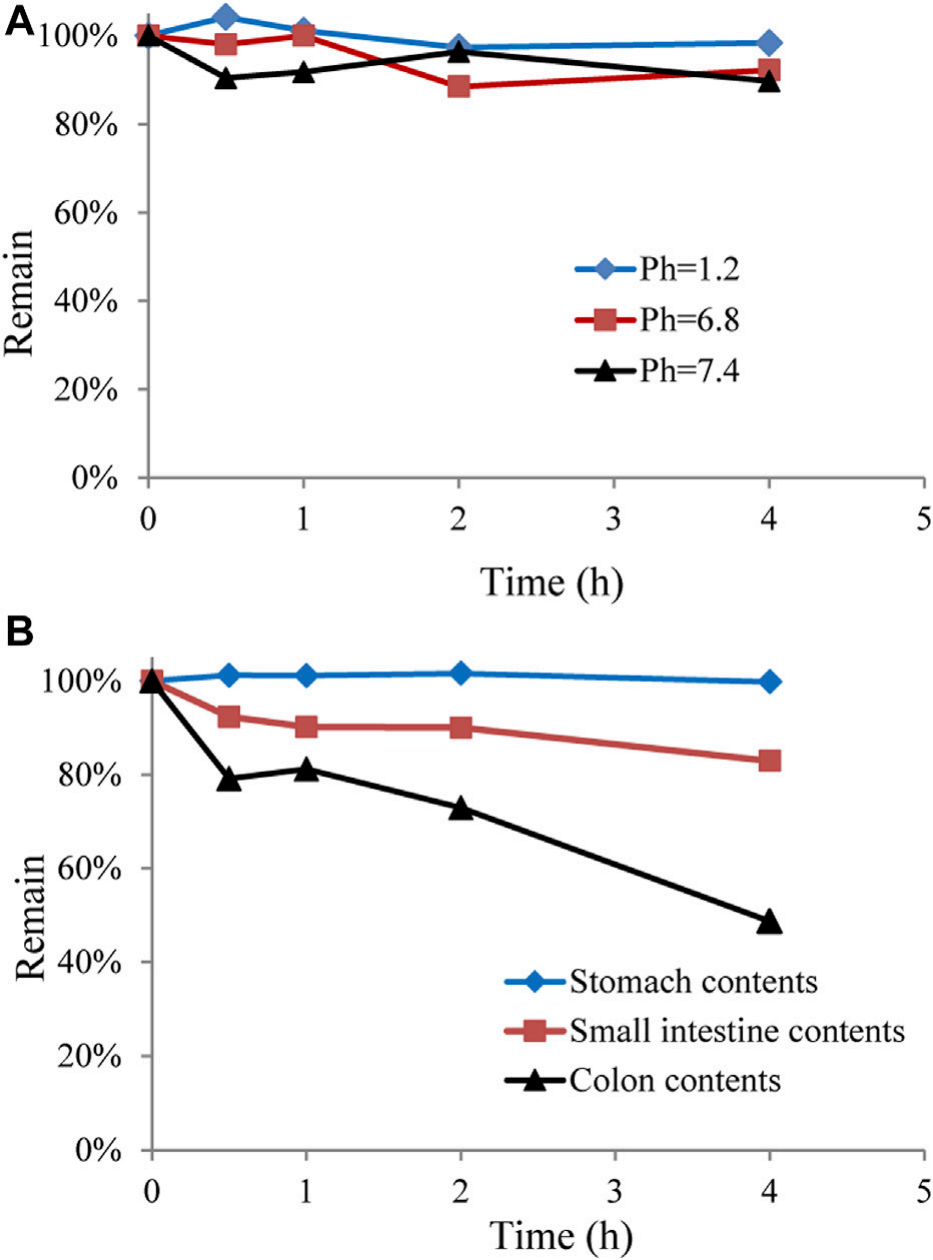
Stability of glycycoumarin in different pH buffer solutions and in gastrointestinal content solutions. Each point represents the mean ± standard deviation (n = 3).

In addition to hepatic metabolism, chemical stability, and drug metabolism in the intestine are considered important factors for low oral bioavailability (Crozier et al., 2009; Stalmach et al., 2012). To further explore whether the low bioavailability of GCM was related to intestinal metabolism, GCM was investigated *in vitro* using different pH buffer solutions and in gastrointestinal content samples. The results indicated that GCM was relatively stable in these tested solutions (pH 1.2, 6.8, and 7.4) and in stomach content solutions after 4 h of incubation at 37°C, but was unstable in the small intestine and colon content solutions (Figure 4). This result is consistent with reports on polyphones, which undergo rapid and extensive first-pass effects via conjugation in the intestinal tract and are poorly bioavailable (Stalmach et al., 2012; Ma et al., 2014; Wang et al., 2014; Wang et al., 2015). Thus, these results indicate the intestinal tract and hepatic first-pass metabolism are the main reasons for low GCM bioavailability.

### 3.5 Tissue distribution

The tissue distribution profile of drugs or compounds *in vivo* is closely related to their pharmacological effects, which determine the strength and duration of these effects. Further, understanding the characteristics of tissue distribution of drugs or compounds is helpful to further understand the sites and extent of their accumulation *in vivo* (Yuan et al., 2015). This will be beneficial in predicting adverse drug reactions, which are significant for providing guidance, especially for certain drugs or compounds metabolized in the body.

**FIGURE 5:**
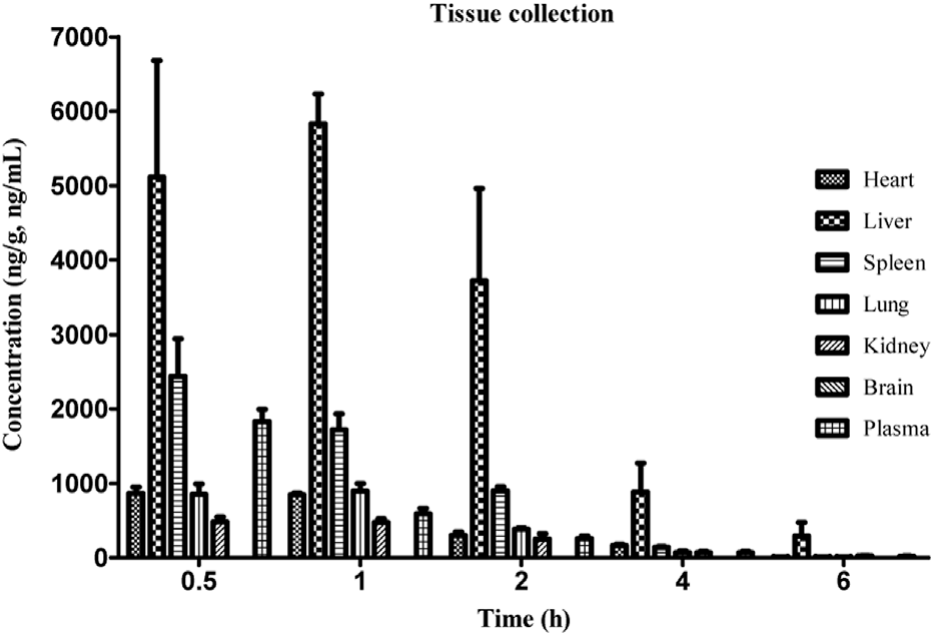
Concentration of glycycoumarin (GCM) in various tissues at the indicated time points after intraperitoneal administration at a single dose of 20 mg/kg to rats. Each point represents the mean ± standard deviation (n = 5).

Our previous studies have shown that intraperitoneal injection of GCM exerts strong hepatoprotective and anti-liver cancer activity (Yan et al., 2018; Zhang et al., 2018). We thus questioned whether GCM is well-distributed in the liver tissue. Combining previous modes of drug administration (Song et al., 2015; Song et al., 2016; Yan et al., 2018), reducing the influence of individual absorption differences in animals, and directly observing the distribution of GCM in rats, intraperitoneal administration was used in the experiments reported here. After intraperitoneal administration of 20 mg/kg, GCM was widely distributed in all tissues, except the brain, as shown in Figure 5. Despite being a lipophilic polyhydroxy compound, GCM was not detected in brain samples. These results indicate that GCM cannot cross the blood-brain barrier. The highest GCM concentration was observed in the liver, followed by that in the spleen, lung, heart, and kidney; the concentration in the liver increased during 0.5–1 h, and then decreased. However, the GCM levels significantly decreased to undetectable levels by 6 h after dosing in most tissues. The highest concentration detected in the liver implied that the liver may be the main binding site for GCM, and that hepatic clearance may represent the primary route for GCM elimination. This may also help explain the hepatic first-pass effect of GCM. The concentration of GCM in plasma decreased rapidly after 0.5 h, which is in agreement with the results of the pharmacokinetic studies. Compared with the plasma concentrations, GCM can be quantified in various tissue samples, meaning that it has a relatively high degree of tissue distribution. Overall, these results provide an important material basis for the pharmacological effects of GCM.

### 3.6 Urinary and biliary excretion of GCM

The total amount of a drug in the circulatory system can be calculated after *i. v.* administration, which can yield the precise excretion rate of a parent drug in bile and urine. However, oral administration cannot result in this because of individual differences in drug absorption. Therefore, we performed *i. v.* administration to explore the excretion rate of GCM. The urinary and biliary recoveries of free and total GCM after *i. v.* administration are displayed in Table 1. The results indicated that a small amount of GCM was excreted as glucuronidated metabolites from bile with approximately 1.97%, 1.71%, 1.51%, and 1.33% of the dose administered over the periods of 0–2, 2–4, 4–6 and 6–8 h post-dosing, respectively. Furthermore, very low amounts of GCM in the parent form were excreted from bile: <1% of the delivered dose over 0–8 h. The urinary recovery of GCM over 24 h after dosing was 0.39% and 0.97% of the administered dose for free and total GCM, respectively. In the study of another polyphenol compound, a large number of sulfonated metabolites can also be excreted through bile and urine in addition to glucuronidated metabolites (Chang et al., 2012). This suggests that biliary and urinary excretion represent minor pathways for glucuronidated GCM metabolites.

The liver is rich in a large number of phase I and II metabolic enzymes, including CYP450, UDP-glucuronosyltransferases (UGTs), and sulfotransferases, which are the main sites of drug metabolism (Zhang et al., 2020). Our results show that a large amount of GCM is distributed in the liver and further metabolized in the liver. In addition to phase I metabolism, parent GCM and its phase I metabolites can undergo glucuronidation reactions by UGT enzymes because there is an abundance of UGT enzymes including UGT1A1, UGT1A3, UGT1A4, UGT1A6, UGT1A9, UGT1A19, and UGT2B7 in the liver (Wang et al., 2014). More recently, GCM was found to strongly inhibit P450 family members, including CYP1A2, CYP2B6, CYP2C8, CYP2C9, and CYP2C19, as well as UGT1A19 in human liver microsomes (Li et al., 2017; Kuang et al., 2021). Furthermore, GCM also significantly inhibits CYP2D6 activity, which mediates the metabolism of approximately 30% of the drugs on the market (Qiao et al., 2014a). Glycyrol, with a similar structure to that of GCM, is another major bioactive coumarin in licorice, as well as being a metabolite of GCM, and is not absorbed into the blood after oral administration (Wang et al., 2015). However, glycyrol also displays competitive inhibitory effects on CYP1A1 and CYP2C9 in human liver microsomes(Kim et al., 2016). In summary, the above results suggest that GCM may cause potential drug interactions when co-administered with agents metabolized by such enzymes. Therefore, as a lead compound with developmental value, the mechanisms of GCM absorption and its inhibitory effects on P450 should be investigated further.

## 4 Conclusion

A simple and reliable LC-MS/MS method for quantifying GCM concentrations in plasma, urine, bile, and tissue samples was developed and validated. To the best of our knowledge, this is the first systematic report regarding the pharmacokinetic and tissue distribution profiles of GCM in rats. The obtained pharmacokinetic and tissue distribution data indicate that GCM can be absorbed by the body with low bioavailability, which is then rapidly converted to conjugated GCM metabolites, and that only a small amount is removed from the body *via* biliary and urinary excretion. Furthermore, GCM is distributed rapidly and widely in various tissues, with the liver representing the major distribution site, whereas GCM does not cross the blood-brain barrier. These results can help us better understand the pharmacological effects of GCM in the body.

## Data Availability

The original contributions presented in the study are included in the article/Supplementary Material, further inquiries can be directed to the corresponding author.
