# Supplementary figures and images for: Absorption, tissue distribution, and excretion of glycycoumarin, a major bioactive coumarin from Chinese licorice (Glycyrrhiza uralensis Fisch)
Source: Front Pharmacol. 2023 Jul 7;14:1216985. doi: 10.3389/fphar.2023.1216985 (PMC10361251; doi:10.3389/fphar.2023.1216985)

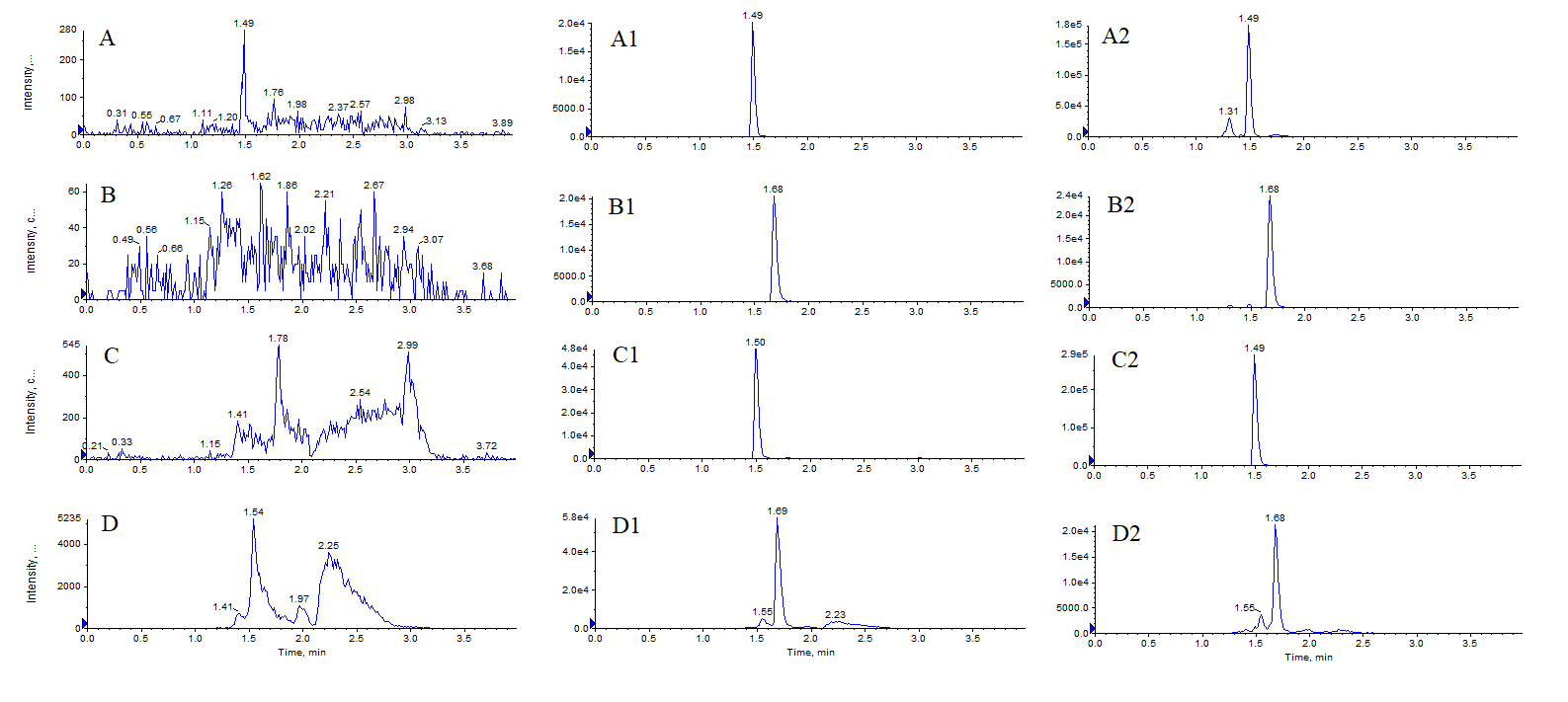

Supplement: Supplementary file 1 [file Image6.TIF]

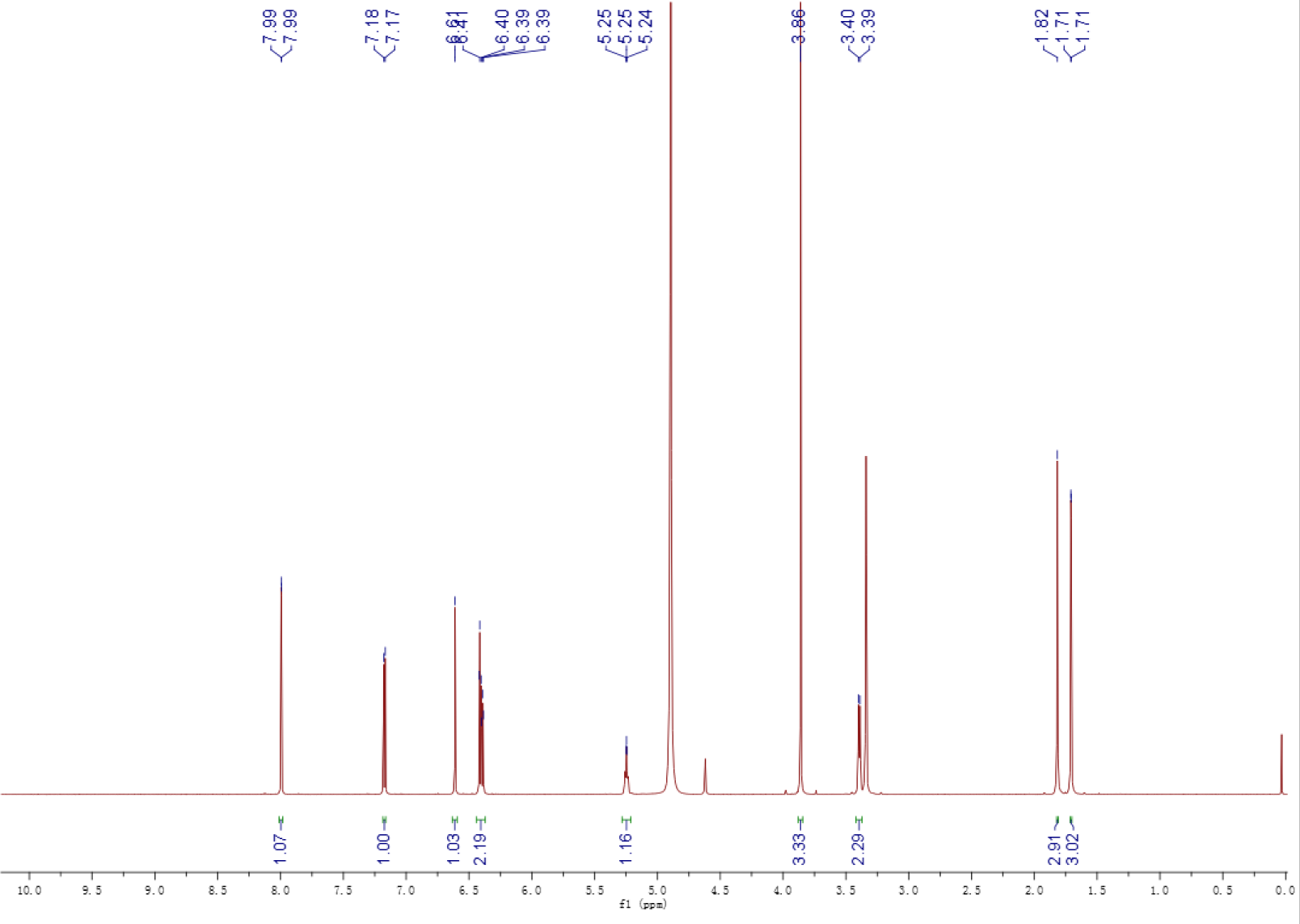

Supplement: Supplementary file 3 [file Image3.TIF]

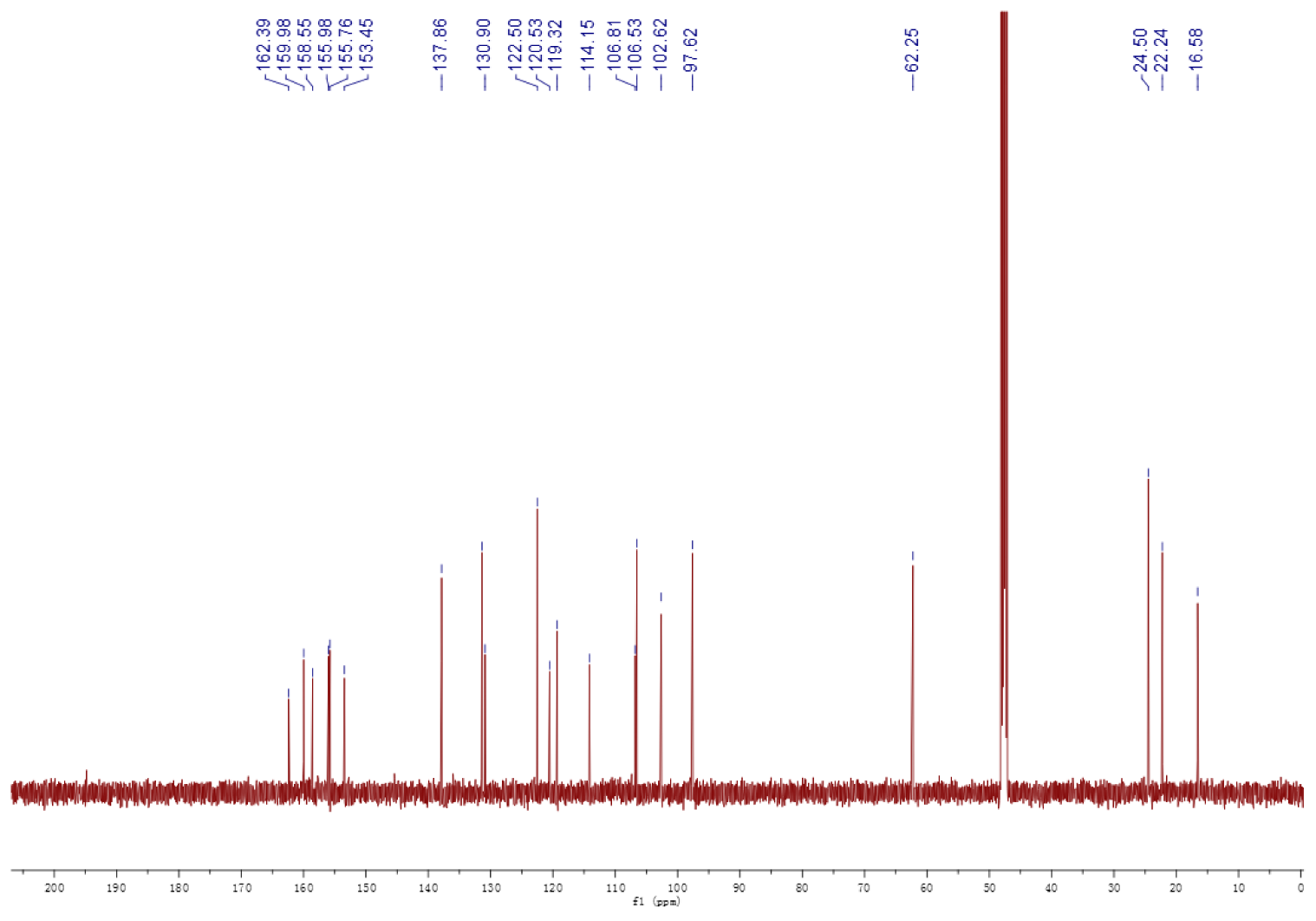

Supplement: Supplementary file 4 [file Image4.TIF]

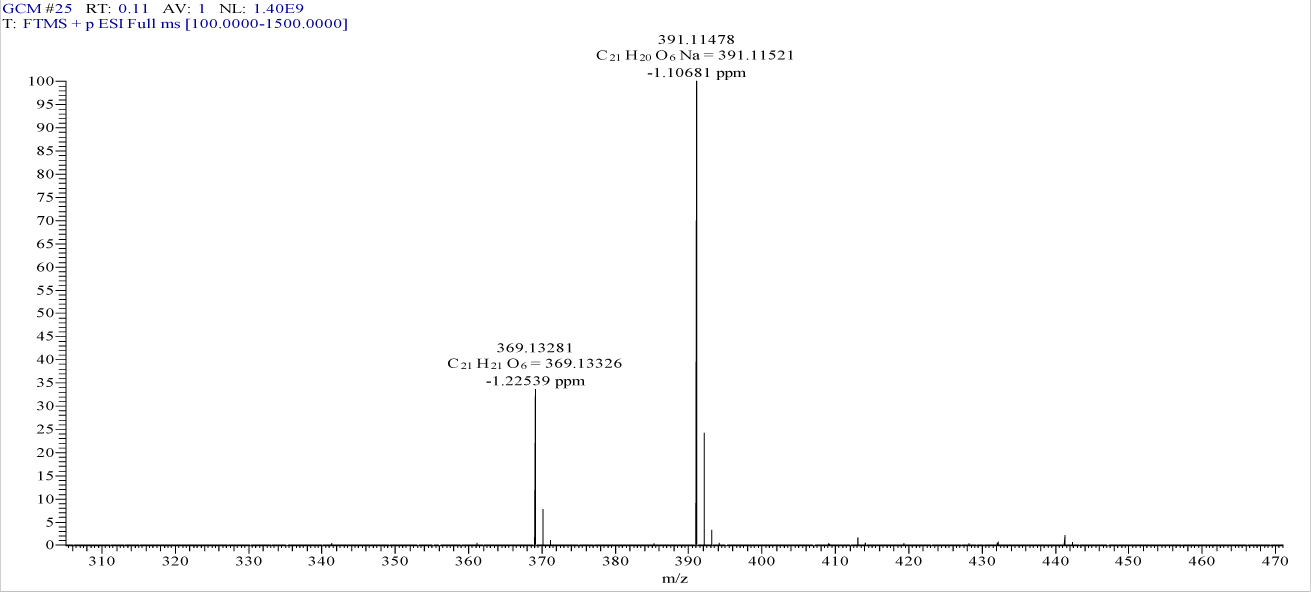

Supplement: Supplementary file 5 [file Image2.TIF]

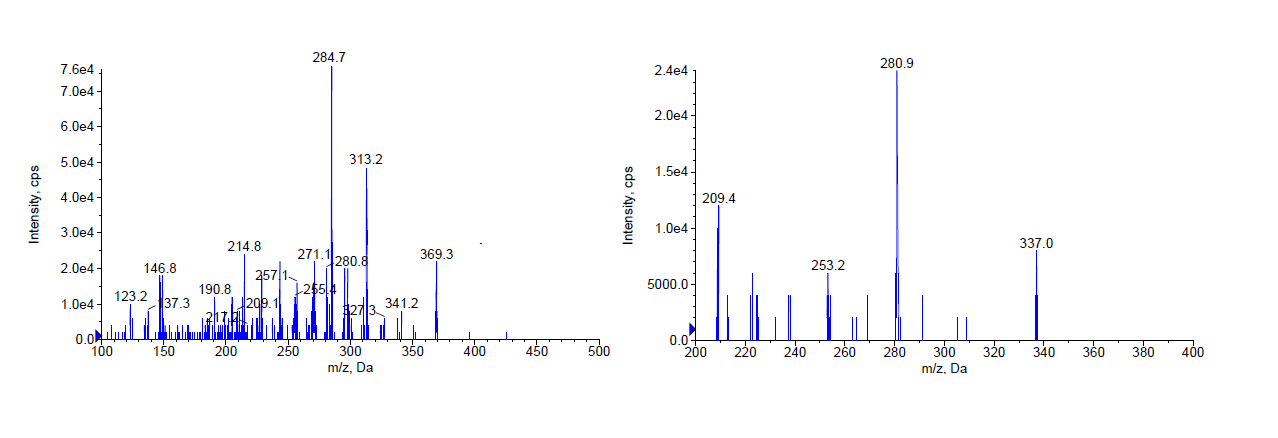

Supplement: Supplementary file 6 [file Image1.TIF]

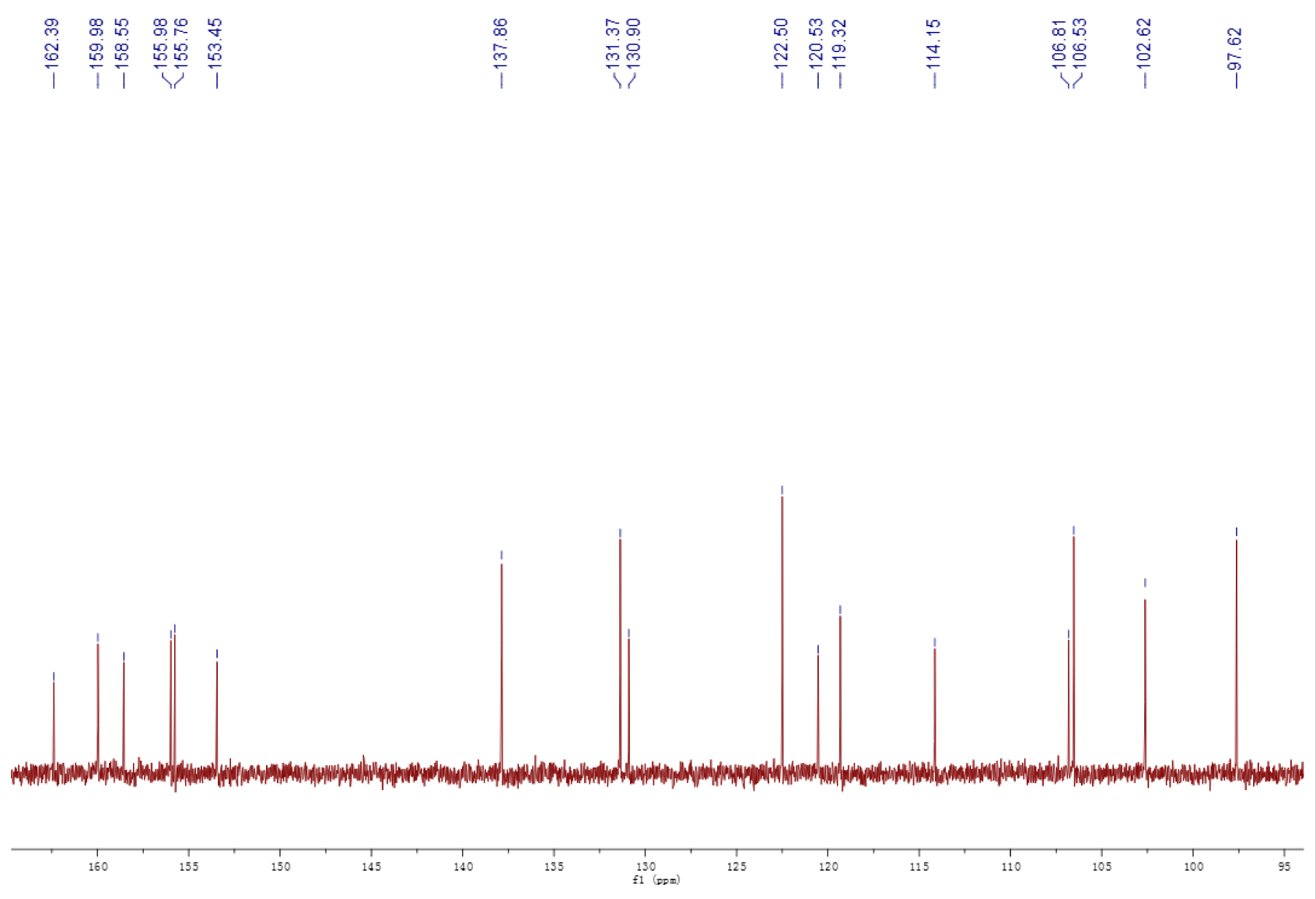

Supplement: Supplementary file 9 [file Image5.TIF]

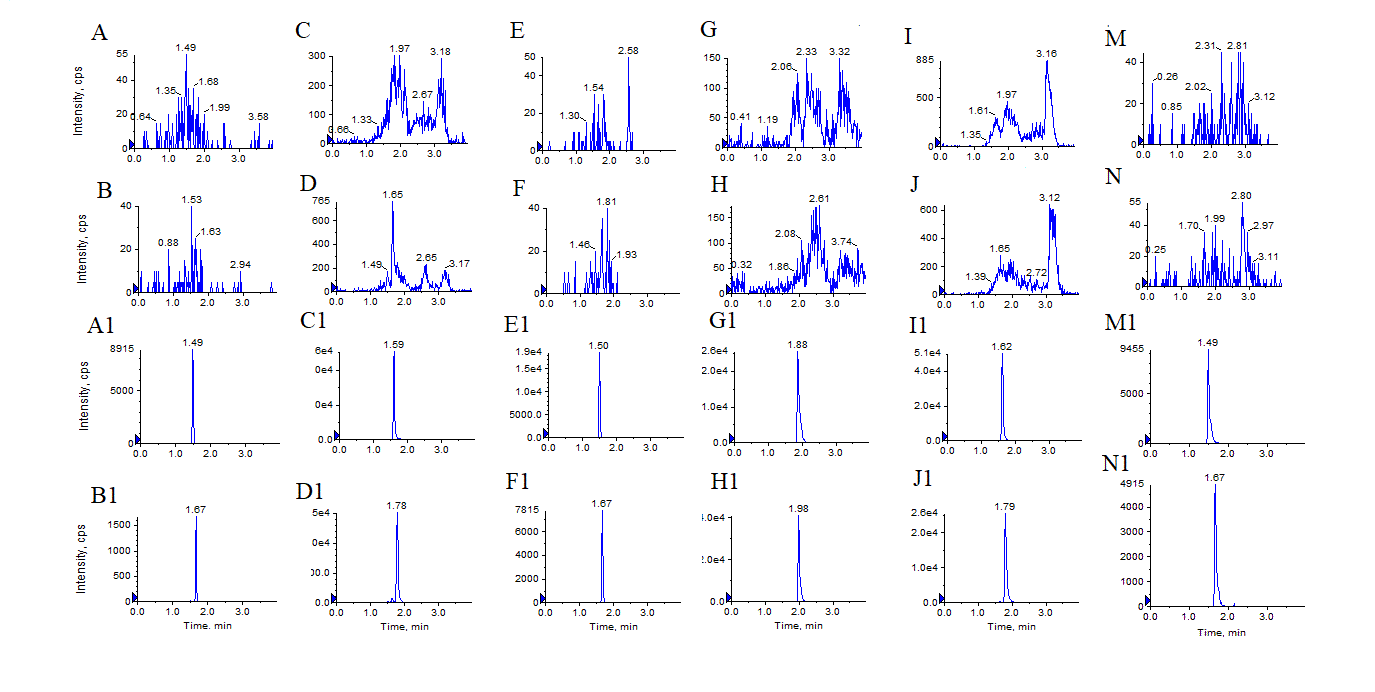

Supplement: Supplementary file 10 [file Image7.TIFF]
