# Supplementary material for: Absorption, tissue distribution, and excretion of glycycoumarin, a major bioactive coumarin from Chinese licorice (Glycyrrhiza uralensis Fisch)
Source: Front Pharmacol. 2023 Jul 7;14:1216985. doi: 10.3389/fphar.2023.1216985 (PMC10361251; doi:10.3389/fphar.2023.1216985)
Supplement: Supplementary file 2 [file Table1.docx]

| sample | Linear range (ng/mL) | Equation | R^2^ | LLOD  (ng/mL) | LLOQ  (ng/mL) |
| --- | --- | --- | --- | --- | --- |
| Plasma | 2-2000 | y = 0.012x + 0.018 | 0.997 | 1 | 2 |
| Bile | 2-2000 | y = 0.005x + 0.004 | 0.998 | 1 | 2 |
| Urine | 2-2000 | y = 0.005x + 0.010 | 0.996 | 1 | 2 |
| Heart | 5-2000 | y = 0.031x + 0.062 | 0.995 | 1 | 5 |
| Liver | 5-2000 | y = 0.009x + 0.009 | 0.998 | 1 | 5 |
| Spleen | 5-2000 | y = 0.018x + 0.066 | 0.973 | 1 | 5 |
| Lung | 5-2000 | y = 0.005x + 0.001 | 0.992 | 1 | 5 |
| Kidney | 5-2000 | y = 0.010x + 0.001 | 0.995 | 1 | 5 |
| Brain | 5-2000 | y = 0.026x + 0.023 | 0.995 | 1 | 5 |
