# Supplementary material for: Absorption, tissue distribution, and excretion of glycycoumarin, a major bioactive coumarin from Chinese licorice (Glycyrrhiza uralensis Fisch)
Source: Front Pharmacol. 2023 Jul 7;14:1216985. doi: 10.3389/fphar.2023.1216985 (PMC10361251; doi:10.3389/fphar.2023.1216985)
Supplement: Supplementary file 7 [file Table2.DOCX]

| sample | QC (ng/mL) | ISNMF (RSD%) | Extraction recovery (%) | Room temperature  (24 h, 4°C) | | Long term  (7 day, -40°C) | |
| --- | --- | --- | --- | --- | --- | --- | --- |
|  |  |  |  | Accuracy (%) | Precision (%) | Accuracy (%) | Precision  (%) |
| Plasma | 15 | 9.42 | 99.91 | 97.99 | 2.12 | 85.53 | 7.87 |
|  | 150 | 12.75 | 94.58 | 97.72 | 4.34 | 103.56 | 2.51 |
|  | 1500 | 5.57 | 88.82 | 99.22 | 6.44 | 99.98 | 4.60 |
| Bile | 15 | 3.57 | 103.3 | 104.47 | 6.23 | 107.39 | 4.26 |
|  | 150 | 1.75 | 102.86 | 101.74 | 6.97 | 109.45 | 6.93 |
|  | 1500 | 2.63 | 107.03 | 97.71 | 4.26 | 110.03 | 8.04 |
| Urine | 15 | 5.43 | 107.43 | 95.60 | 8.35 | 114.13 | 8.99 |
|  | 150 | 3.49 | 99.49 | 104.48 | 4.07 | 114.02 | 2.35 |
|  | 1500 | 7.06 | 107.11 | 103.42 | 1.73 | 111.16 | 7.50 |
| Heart | 15 | 3.49 | 106.45 | 111.55 | 8.04 | 103.22 | 8.97 |
|  | 150 | 9.50 | 85.47 | 107.11 | 7.89 | 105.75 | 2.62 |
|  | 1500 | 4.34 | 104.47 | 107.22 | 6.74 | 109.39 | 5.06 |
| Liver | 15 | 7.1 | 98.19 | 85.45 | 11.46 | 111.11 | 10.10 |
|  | 150 | 4.22 | 101.84 | 106.63 | 5.07 | 99.36 | 13.98 |
|  | 1500 | 6.38 | 101.21 | 112.23 | 10.74 | 101.15 | 4.22 |
| Spleen | 15 | 9.27 | 110.91 | 97.83 | 14.48 | 99.34 | 7.98 |
|  | 150 | 8.91 | 89.50 | 98.25 | 2.95 | 101.12 | 5.99 |
|  | 1500 | 9.35 | 86.82 | 104.83 | 8.47 | 101.54 | 6.96 |
| Lung | 15 | 9.56 | 95.12 | 99.50 | 6.92 | 108.78 | 10.09 |
|  | 150 | 4.06 | 111.34 | 102.29 | 5.08 | 97.62 | 5.41 |
|  | 1500 | 11.21 | 107.29 | 103.30 | 3.96 | 112.19 | 10.15 |
| Kidney | 15 | 12.65 | 100.07 | 98.45 | 14.18 | 90.87 | 8.91 |
|  | 150 | 9.72 | 88.91 | 104.34 | 5.12 | 88.95 | 3.15 |
|  | 1500 | 9.08 | 91.63 | 100.19 | 2.69 | 85.88 | 1.64 |
| Brain | 15 | 13.58 | 95.32 | 88.13 | 12.61 | 91.94 | 7.01 |
|  | 150 | 14.14 | 111.54 | 98.45 | 4.09 | 96.69 | 2.75 |
|  | 1500 | 10.87 | 104.31 | 107.49 | 3.43 | 102.47 | 5.51 |
| ISNMF, internal standard normalized matrix | | | | | | | |
