# Supplementary material for: Absorption, tissue distribution, and excretion of glycycoumarin, a major bioactive coumarin from Chinese licorice (Glycyrrhiza uralensis Fisch)
Source: Front Pharmacol. 2023 Jul 7;14:1216985. doi: 10.3389/fphar.2023.1216985 (PMC10361251; doi:10.3389/fphar.2023.1216985)
Supplement: Supplementary file 8 [file Table3.DOCX]

| sample | QC (ng/mL) | Intra-day | | Inter-day | |
| --- | --- | --- | --- | --- | --- |
|  |  | Accuracy (%) | recision (RSD%) | Accuracy (%) | Precision (RSD%) |
| Plasma | 5 | 93.51 | 6.83 | 102.69 | 14.91 |
|  | 15 | 93.14 | 4.69 | 90.31 | 4.14 |
|  | 150 | 102.97 | 1.37 | 89.30 | 1.35 |
|  | 1500 | 100.57 | 2.97 | 86.31 | 2.96 |
| Bile | 5 | 92.77 | 13.42 | 91.92 | 12.65 |
|  | 15 | 93.78 | 3.55 | 97.36 | 7.60 |
|  | 150 | 91.10 | 2.70 | 92.45 | 2.85 |
|  | 1500 | 90.09 | 1.72 | 90.54 | 2.29 |
| Urine | 5 | 111.00 | 9.64 | 91.73 | 11.37 |
|  | 15 | 98.04 | 6.02 | 118.42 | 3.01 |
|  | 150 | 97.29 | 6.62 | 114.82 | 2.72 |
|  | 1500 | 87.73 | 3.25 | 113.36 | 3.69 |
| Heart | 15 | 103.64 | 12.25 | 88.59 | 9.14 |
|  | 150 | 99.11 | 5.54 | 96.56 | 4.98 |
|  | 1500 | 101.29 | 8.10 | 85.69 | 10.25 |
| Liver | 15 | 88.86 | 7.99 | 92.75 | 7.59 |
|  | 150 | 105.28 | 4.44 | 101.00 | 3.93 |
|  | 1500 | 100.8 | 3.43 | 92.42 | 1.86 |
| Spleen | 15 | 95.15 | 14.02 | 106.47 | 13.23 |
|  | 150 | 114.06 | 4.26 | 113.47 | 5.51 |
|  | 1500 | 109.58 | 4.32 | 88.36 | 2.12 |
| Lung | 15 | 111.39 | 12.61 | 105.65 | 9.54 |
|  | 150 | 105.94 | 4.93 | 104.00 | 4.55 |
|  | 1500 | 107.4 | 4.25 | 104.06 | 4.40 |
| Kidney | 15 | 94.86 | 8.38 | 97.04 | 11.43 |
|  | 150 | 113.33 | 2.82 | 110.53 | 4.34 |
|  | 1500 | 105.93 | 5.67 | 105.93 | 3.90 |
| Brain | 15 | 88.13 | 12.61 | 114.23 | 6.83 |
|  | 150 | 98.45 | 4.09 | 99.05 | 5.19 |
|  | 1500 | 107.49 | 3.43 | 100.31 | 3.80 |
